# Perturbations in the T cell receptor β repertoire during malaria infection in children: A preliminary study

**DOI:** 10.3389/fimmu.2022.971392

**Published:** 2022-10-13

**Authors:** Augustina Frimpong, Michael Fokuo Ofori, Abdoelnaser M. Degoot, Kwadwo Asamoah Kusi, Buri Gershom, Jacob Quartey, Eric Kyei-Baafour, Nhi Nguyen, Wilfred Ndifon

**Affiliations:** ^1^ West African Centre for Cell Biology of Infectious Pathogens (WACCBIP), Department of Biochemistry, Cell, and Molecular Biology, University of Ghana, Accra, Ghana; ^2^ Immunology Department, Noguchi Memorial Institute for Medical Research, College of Health Sciences, University of Ghana, Accra, Ghana; ^3^ African Institute for Mathematical Sciences, Accra, Ghana; ^4^ Research Department, African Institute for Mathematical Sciences, Next Einstein Initiative, Kigali, Rwanda; ^5^ African Institute for Mathematical Sciences, Cape Town, South Africa; ^6^ MIODX Inc, San Jose, CA, United States

**Keywords:** malaria, T cell receptor (TCR), repertoire, diversity, sequencing, public clonotypes

## Abstract

The changes occurring in the T cell repertoire during clinical malaria infection in children remain unknown. In this study, we undertook the first detailed comparative study of the T cell repertoire in African children with and without clinical malaria to test the hypothesis that clonotypic expansions that occur during *P. falciparum* infection will contribute to the generation of a T cell repertoire that is unique to each disease state. We profiled the complementarity-determining region 3 (CDR3) of the TCRβ chain sequences from children with *Plasmodium falciparum* infections (asymptomatic, uncomplicated and severe malaria) and compared these with sequences from healthy children. Interestingly, we discovered that children with symptomatic malaria have a lower TCR diversity and frequency of shared (or “public”) TCR sequences compared to asymptomatic children. Also, TCR diversity was inversely associated with parasitemia. Furthermore, by clustering TCR sequences based on their predicted antigen specificities, we identified a specificity cluster, with a 4-mer amino acid motif, that is overrepresented in the asymptomatic group compared to the diseased groups. Further investigations into this finding may help in delineating important antigenic targets for vaccine and therapeutic development. The results show that the T cell repertoire in children is altered during malaria, suggesting that exposure to *P. falciparum* antigens disrupts the adaptive immune response, which is an underlying feature of the disease.

## Introduction

Malaria is an infectious disease caused by protozoans from the genus *Plasmodium.* According to WHO, there was no significant reduction in the incidence of the disease in 2018 when compared to 2017 (1). In addition, there is no licensed effective vaccine meeting the efficacy targets set in the Malaria Vaccine Technology Roadmap (2). Natural infections in malaria-endemic areas may result in anti-disease or anti-parasite protection but not sterile immunity (3–5). The magnitude of host immunity is in large part determined by the activity of T cells, which are central to the immune activity of other immune cells (6, 7).

Therefore, antigen-specific T cells in malaria may play crucial roles in defining the disease status. This specificity of T cells is determined by the T cell receptor (TCR), which is generated during T cell development in the thymus. The TCR is heterodimeric, consisting of either an αβ (involved in adaptive response) or γδ (mostly involved in innate response) chains (8, 9). The β chain, unlike the α chain, consists of a diversity (D) gene segment in addition to variable (V) and joining (J) gene segments resulting in a V(D)J instead of a VJ for the α chain. These gene segments are combined through a recombination event that occurs in the complementarity determining region 3 (CDR3) of the β chain (10). During the recombination, the diversity (D) region enhances specificity of the TCR through the addition and deletion of nucleotides in the junctional regions between the gene segments. This further ensures the recognition of parasite-specific antigens and effective T cell responses against a diverse range of pathogen-derived antigens. Therefore, profiling the CDR3 sequences (TCR repertoire) can provide insight into a person’s infection history as well as the progression or outcome of new infections. Besides, if we adopt the common definition of TCR diversity based on the Simpson’s Diversity index, D (11), then it follows that a preferential increase in frequency of a subset of TCRs will lead to a decline in TCR diversity, which can help define an individual’s immune competence.

Indeed, previous work has documented changes in TCR repertoires occurring in various human clinical conditions such as autoimmune diseases (12, 13), infectious diseases (14–16), and ageing (17). For instance, in tuberculosis, limited diversity has been correlated with severe disease progression (18). However, for malaria, studies on the TCR repertoire have focused on murine models of infection which mostly does not reflect human malaria infections (19–21). Nonetheless, these studies have revealed an increased frequency of specific Vβ gene segments. In some of these animal model studies, pathogenic roles have been ascribed to the Vβ8.1 segment, an immune receptor gene that reacts with *P. berghei-*specific antigen (19, 22) believed to be crucial for the pathogenesis of cerebral malaria.

However, whether this is similar in human clinical infections remains to be investigated. Therefore, in this preliminary study, we profiled the TCR β repertoire in children with *P. falciparum* infections using high throughput sequencing. We hypothesized that clonotypic expansions that occur during *P. falciparum* infection will contribute to the generation of a TCR repertoire that is unique to each disease state. Therefore, PBMCs obtained from 28 children with or without *P. falciparum* infection were sequenced.

## Materials and methods

### Study design and cohort

This study formed part of an earlier cross-sectional study that characterized T cell phenotypic responses in children with *P. falciparum* infections (5, 23). Blood samples were collected from children residing in a hyperendemic area for malaria as previously described Exclusion criteria for the study were children with evidence of concomitant infection including bacterial infections, history of an underlying disease including sickle cell disease, and those who had taken anti-malaria drugs two (2) weeks preceding the study recruitment. The inclusion criteria for enrollment were presence of parasitemia in blood, no axillary temperature of ≤ 37.5°C or any other ailment (for asymptomatic children); axillary temperature >37.5 °C, presence of parasitemia, no other sources of underlying ailment (for uncomplicated malaria children); high parasitaemia, fever, Blantyre coma score ≤ 3, respiratory distress, ≥2 seizure episodes in 24 hours (for severe malaria); healthy controls were children with no fever nor infection. Participants were recruited after informed consent and assent were received from guardians and the children respectively. For this study, a total of 28 children were included, categorized as follows: healthy controls (n=4), asymptomatic (n=7), uncomplicated malaria (n=9), severe malaria (n=8). Children with clinical malaria were recruited from the health facilities whereas asymptomatic and control children were recruited from households within the community. All participants were children under 12 years of age (**Table 1**). Study participants were recruited from a hyper-endemic malaria transmission zone in Ghana.

**Table 1:** Characteristics of the study population.

Characteristics	Controls	Asymptomatic	Uncomplicated	Severe malaria
Sample size (n)	4	7	9	8
Age (IQR), years	9.5 (7.5-10)	7 (2-9)	8.5 (7.25-10.5)	4.5 (2.25-7)
Female (n)	1	3	3	4
Parasitemia (IQR), µl	NA	1,762 (621.4–4,193)	26,357 (7,143–28,341)	47,713 (7,777–67563)
Productive sequences	6157	355177	192424	40725

IQR, interquartile range; NA, not applicable,

### Sample collection

Children included in this study were sub-sampled from the cohorts described earlier. Briefly, about 5 ml of venous blood was obtained from the participants into heparin tubes before anti-malarial treatment. For this current study, the presence of malaria parasites was determined using polymerase chain reaction (PCR) and Giemsa-stained thick blood smears. The PCR was used to exclude any other Plasmodium infections except falciparum as well as to exclude children with submicroscopic infections in the control group. Parasite densities were estimated using Giemsa-stained thick blood smears by counting the number of *P. falciparum* parasites per 200 white blood cell counts (24).

Children with asymptomatic malaria were then defined by the presence of *P. falciparum* microscopic infection with no clinical symptoms whereas healthy controls were defined by the absence of both microscopic and submicroscopic *P. falciparum* infections. Peripheral Blood Mononuclear Cells (PBMCs) were isolated by Ficoll gradient centrifugation. About 1 x 105 cells were stored in trizol for RNA isolation.

### Library preparation and sequencing

Library preparation and sequencing were performed according to the miodx Clonomap™ immune repertoire sequencing assay using the Illumina MiSeq platform (miodx, San Hose, CA). Total RNA was extracted from PBMC obtained from malaria positive and negative controls. For the library preparation, first strand cDNA synthesis was performed with template switch adapters using about 50-2000 ng of starting mRNA and 20% v/v TCRβ primer specific for the constant region. The cDNA libraries generated were amplified using two PCR amplification procedures. The first PCR amplification was performed using 44% v/v of purified cDNA products under the following conditions: 94°C for 60 secs for 1 cycle and 98°C for 10 secs for denaturing for 20 cycles, 60°C for 15 secs annealing and 68°C for 10 secs for extension for 20 cycles respectively. The second PCR amplification was performed using the same conditions but 16 cycles for the denaturing, annealing and extension. The PCR products obtained were loaded on a 2% agarose gel with SYBR green staining. Bands within the size ranges of 500-800 bp were excised and purified before library quantification. The libraries were paired-end sequenced using an Illumina MiSeq platform.

### Bioinformatic analysis

The sequencing data were de-multiplexed, quality-filtered, and clustered based on the unique molecular identifiers to accurately quantify the frequencies of unique TCR clones. The V, D and J genes segments of each uniquely identified clonal sequence were determined by alignment to germline sequences found in the ImMunoGeneTics database (25). The nucleotide sequences were subsequently translated into amino acid sequences. For VDJ annotation, CDR3 regions were identified as the subsequence occurring between the last conserved cysteine found at the 3’ end of the Vβ and the conserved phenylalanine found at the 5’ end of the Jβ gene segment. CDR3 sequences with stop codons were classified as out-of-frame or non-productive sequences.

### Estimating TCR repertoire diversity

TCR repertoire diversity was estimated using the Renyi’s entropy (26). Renyi’s entropy unifies various diversity indices into a diversity profile characterized by an order parameter, α. Mathematically, the Renyi’s entropy of a given TCR repertoire *X* is defined as:


1 $$H_{\alpha }(X)=\frac{1}{1-\alpha }\text{log(}\sum_{x\in X}{\left(f_{x}\right)}^{\alpha })$$

where α≠1, f_x_(*x*) is the frequency of TCR *x*, and the sum is over all distinct TCRs *x* in *X*.

When α=0, the Renyi entropy equals the number of distinct TCRs found in the TCR repertoire under consideration.This is considered as the richness of the repertoire. In the limit as α tends to 1, the Renyi entropy equals the exponent of the famous Shannon entropy used in information theory. Whereas, when α=2, the Renyi entropy equals the reciprocal of the famous Simpson’s index used to estimate diversity in ecology, BCR and TCR repertoires.

### Estimating TCR repertoire divergence

The Kullback-Leibler divergence (KLD) was used to estimate the difference between the distributions of V, J and VJ combinations found between the study groups. For example, in the case of V gene segments, the KLD between the two groups with V distributions given by *g* and *h*, respectively, is defined as:


2 $$\text{KLD(}g||h)=\sum_{v}g(v)\text{log(}\frac{g(v)}{h(v)})$$

*g(v*) and *h*(*v*) denote the frequency of segment *v* in groups 1 and 2, respectively.

Bootstrap samples of the V, J, and VJ segments were generated under the null hypothesis that there were no differences between the considered groups. KLD values were also calculated based on the bootstrap samples. The proportion of these bootstrap KLD values that were lower than or equal to the corresponding real KLD value was determined and used to determine statistical significance of the latter value at a false discovery rate (27) of 5%.

### Identification of public and private TCRs

CDR3 amino acid sequences were designated as public if they satisfied one of two criteria: 1) they occurred in all individuals in a group, or 2) they occurred in at least 75% of the individuals in a group. Additionally, a CDR3 amino acid was termed private when exclusively expressed in a single individual.

### Identification of T cell receptors with similar antigen specificity

We identified groups of TCRs with similar antigen specificity using the GLIPH algorithm developed by Glanville et al. (28), which clusters TCR sequences based on their global and local similarities. Briefly, global similarity identifies TCR sequences that differ in at most one amino acid. In contrast, local similarity identifies TCRs that share CDR3 motifs (2-mers, 3-mers, 4-mers) which are statistically enriched in pathogen-exposed versus naïve TCR repertoires.

### Identifying target antigens of clustered TCR motifs using VDJdb

The VDJ database (db) is a curated database that has unique TCR sequences with their corresponding antigenic epitopes and MHC classes. TCRβ sequences were retrieved and matched to determine the target epitopes to which there is a predicted reactivity (29). First, CDR3 sequences from VDJdb that were matched with our sequences and shared the same V and J gene segments were identified. Secondly, CDR3 sequences that differed by one amino acid mismatch but shared the same V and J gene segments were also identified.

### Statistical analysis

The students’ t-test and ANOVA (for normally distributed data) and Mann-Whitney or Kruskal-Wallis test (for non-normally distributed data) were used to assess the significant differences amongst the groups. Benjamini-Hochberg’s multiple comparison test was used to correct for multiple testing at a false discovery rate of 5%. Correlation was performed with the Spearman’s rank correlation coefficient. Proportions were assessed for significance using the Chi-square test. Graphs were developed with R statistical software (version 3.4.8, R Development Core Team) and GraphPad Prism (version 6.01 GraphPad Software, Inc.). P values were considered significant at p< 0.05.

## Results

### Summary characteristics on study population

To determine the TCR repertoire in malaria infections, 28 children were sampled: 4 healthy control children, 7 asymptomatic, 9 uncomplicated and 8 severe malaria cases. The characteristics of the study populations are summarized in **Table 1**. The median age of children with severe malaria was lower when compared to the other groups. The median levels of parasitemia were highest in the severe malaria group and lowest in the asymptomatic group. The total number of sequences differed among the groups and was highest in the asymptomatic group.

### The CDR3 length distribution is skewed towards shorter fragments in P. falciparum infected children

**Figure 1:**
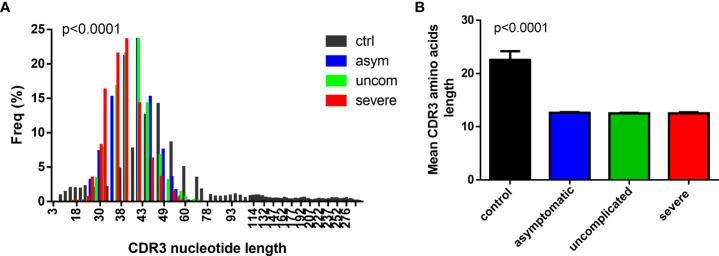
Decreased CDR3 length in *P. falciparum* infected group. A histogram plot showing the comparison of the **(A)** CDR3 length distribution for the **(A)** control (n=4), asymptomatic (n=7), uncomplicated (n=9) and severe groups (n=8), and **(B)** the mean CDR3 aa length for the controls, asymptomatic uncomplicated and the severe malaria groups.

The CDR3 region of the TCR is highly polymorphic and important in determining the diversity of the TCR repertoire. Longer CDR3 lengths/loops have been associated with increased potential to show sequence variation and with the capacity to reach into antigenic pockets (30–32). To assess the CDR3 length distribution in the TCR repertoire, we first determined whether the data conformed to a Gaussian distribution (33). It was observed that the CDR3 length distribution for the diseased states, even though Gaussian-like, was left skewed compared to the controls (**Figure 1A**). Additionally, the CDR3 lengths in the *P. falciparum* infected groups were shorter compared to the healthy controls. (**Figure 1B**). In addition, the frequency of the CDR3 nucleotide length for the controls peaked at the 48 nucleotide sequences; for the asymptomatic and uncomplicated groups, the lengths peaked at a much shorter length of42 nucleotides and it was even shorter for the severe cases (39 nucleotides).

### Differential usage of the Vβ and Jβ gene segments across malaria disease states

**Figure 2:**
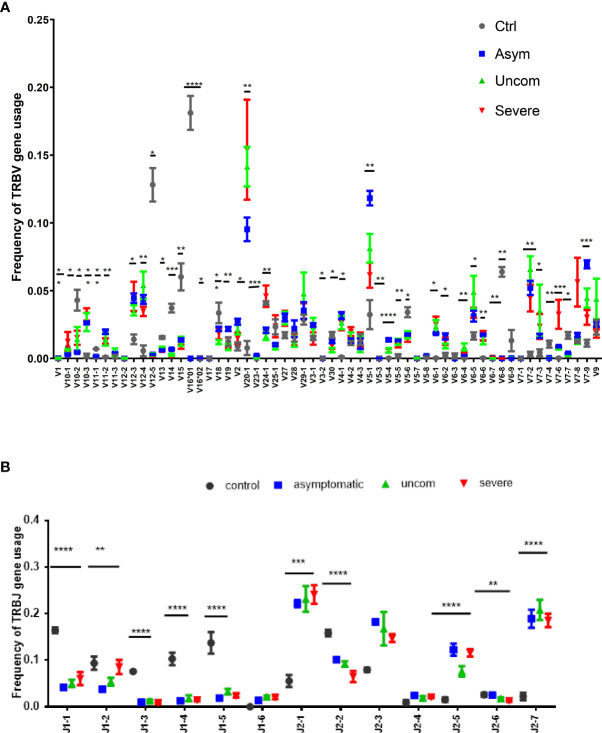
Pattern of Vβ and Jβ gene usage. The data show the frequency of **(A)** Vβ and **(B)** Jβ gene usage among the study population; controls (n=4), asymptomatic (n=7), uncomplicated (n=9) and severe malaria (n=8). The plots show the mean proportions with the standard error. The asterisks (*) show the significant difference in the gene usage; ****p<0.0001, ***p<0.001, **p<0.01, *p<0.05. One-way ANOVA was used for comparisons followed by Holm Sidak’s correction for multiple testing.

The recombination of the V, D and J gene segments generates the TCR clonotypes, whose frequencies are subsequently shaped by encounters with antigens that may lead to biases in the selection of the V and J gene regions. Here, we determined whether the distributions of the V and J gene segments produced during Plasmodium infections are skewed. We identified 59 unique T cell receptor β variable (TRBV) gene segments and 13 unique TRBJ gene segments. The pattern of usage of the Vβ and Jβ gene segments for each group is shown in **Figure 2** and in **Supplementary Figures 1**, **2**.

The pattern of usage of the Vβ gene segments was non-uniform for all the study groups. However, among the *P. falciparum* infected groups, Vβ 20-1 and Vβ5-1 gene segments were predominant. Overall, the usage of over 30 gene segments were significantly different between the infected and the control group (p<0.05). For the TRBJ gene segments, Jβ2 was dominant in all the *P. falciparum-*infected groups compared to the control group. Together, these results imply that *P. falciparum* may modulate the T cell repertoire through the selective use by T cells of specific Vβ and Jβ gene segments for recognition of *P. falciparum* antigens.

### Selective usage of Vβ and Jβ gene combinations during *P. falciparum* malaria

**Figure 3:**
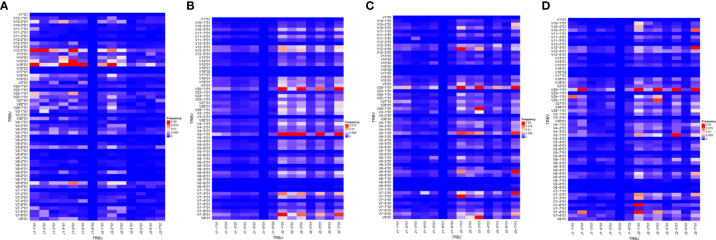
Frequency of V and J gene pairing. The VJ repertoire in each group **(A)** control (n=4); **(B)** asymptomatic (n=7); **(C)** uncomplicated (n=9);**(D)** severe malaria (n=8) group were plotted. The X and Y axes list all possible V and J gene combinations displaying the frequency of usage; with red being the highest frequency, blue representing the lowest frequency.

The skew observed in the distributions of V and J gene segments found in children with malaria was also observed in the distribution of VJ combinations (**Figure 3**). Among the infected group, TRBV20 and TRBJ2 (2-1, 2-3, 2-5 and 2-7) gene segments were mostly found together. Within the infected groups, the combination of TRBV5-1 and TRBJ2 segments was more prevalent in asymptomatic cases compared to uncomplicated malaria cases. In severe cases, T cells preferentially used the combination of TRBV7-8 and TRBV7-6 segments with J2 segments, especially J2-1 and J2-7. The VJ combinations found in the healthy controls were mostly between TRBV12-5/16 and J1 gene segments, with the most prominent being between TRBV 16 and TRBJ2-2. These data suggest a strong clonal selection of some TRBV and TRBJ gene segment combinations in each disease state. The global usage of the VJ combinations of each group of the asymptomatic and uncomplicated groups are shown in **Figure 3**. Interestingly, we also observed that the usage of the TRBV 16*01-J1-1; V12-5*01-J1-5; V15-J1-5; V16*01-J2-2 segment combinations segregated children without malaria from the malaria-infected group (**Supplementary Figure 3**)

### Increased TCR diversity during asymptomatic *P. falciparum* infection

**Figure 4:**
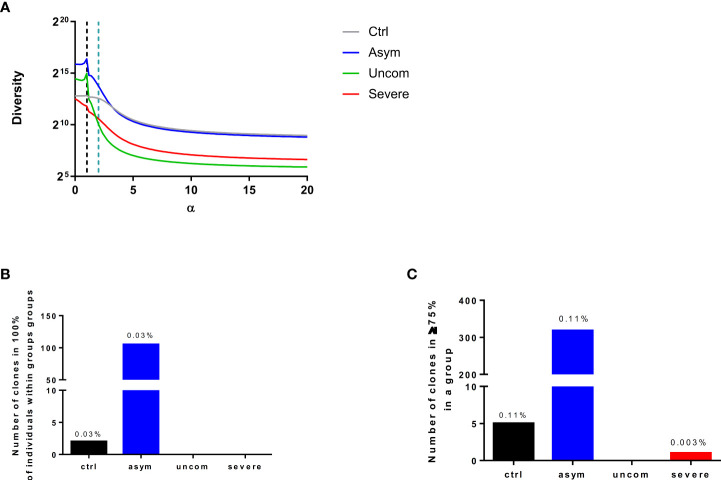
TCR diversity and sharing is increased during asymptomatic *P. falciparum* infection. **(A)** The diversity of the CDR3 measured using Renyi’s entropy. The black dotted lines indicate the Shannon’s entropy and the blue dotted lines indicate the Simpson’s index of diversity. The graphs show the number of CDR3 sequences shared within **(B)** 100% **(C)** at least 75% of individuals in each group.

The TCR diversity depends on the species richness and relative abundance of the clonotypes, which in turn shape the T cell response (34, 35). Diversity is likely to be affected by clonal expansion after antigenic stimulation. To determine the diversity, we used Renyi’s entropy which is based on Hill’s diversity profiles. Renyi’s entropy depicts a continuum of diversity indices based on a range of α values. At smaller αvalues, the diversity estimate accords a greater weight to low-abundance TCRs (increased richness) than it does at larger αvalues (relative abundance). In fact, at larger αvalues the diversity estimate is determined almost exclusively by the clonally expanded TCRs. Comparison of the infected groups yields some striking observations. Firstly, all the infected repertoires have a relatively high TCR richness, ranging from 5870.88 TCRs in the severe malaria group to 59870.3 TCRs in the asymptomatic malaria group. As αincreases from 0 to 20, the diversity estimates declines in all groups (**Figure 4A**), as it accounts for increasingly smaller subsets of TCRs with high frequencies. In all these TCR subsets, diversity is higher in the asymptomatic group compared to the other groups. In contrast, while richness is higher in the uncomplicated malaria group compared to the severe malaria group, the diversity of clonally expanded TCRs is higher in the severe group (**Figure 4A**). Interestingly, for the most inclusive estimates of diversity (corresponding to αvalues of 0-1.6), diversity was inversely correlated with parasitemia (Spearman’s correlation; -0.4794 — -0.5223; p<0.05, **Table 2**).

**Table 2:** Significant correlation between diversity and parasitaemia in *P. falciparum* infected group.

Alpha-values	Spearman’s correlation
0	-0.5099
0.2	-0.5223
0.4	-0.5155
0.6	-0.5076
0.8	-0.5065
1.0	-0.4805
1.2	-0.502
1.4	-0.485
1.6	-0.4794

### Decreased frequency of public TCRs during malaria

TCRs are termed “public” when they are found in many individuals and termed “private” when they are restricted to an individual. The discovery of public TCRs has attracted significant interest because their existence is stochastically unexpected. Given the fact that the number of potential TCRs is larger, by many orders of magnitude, than the number of TCRs that any individual can harbor, stochastic generation of TCRs would make the probability of finding the same TCR in multiple individuals negligible. Nevertheless, public TCRs are common and they have been implicated in immune responses to human immune deficiency virus (HIV) (36, 37), cytomegalovirus (CMV) (38) and other pathogens. To determine whether public TCRs are associated with any of our study groups (healthy, asymptomatic, uncomplicated or severe malaria), we searched for TCRs that are common within and across the groups. We determined the number of TCRs that occur in either all or at least 75% of individuals within a group. The former TCRs were denoted “fully public” whereas the latter were denoted “partially public”. We found that both the control and the asymptomatic groups contained a comparable frequency of fully public TCRs (0.03%), whereas such TCRs were absent from the uncomplicated malaria and severe malaria groups. In addition, 0.11% of the TCRs present in the control and asymptomatic groups were partially public, whereas only a single partially public TCR (0.003%) was found in the severe malaria group (**Figures 4B, C**).

### Convergent recombination events in the asymptomatic T cell repertoire

**Figure 5:**
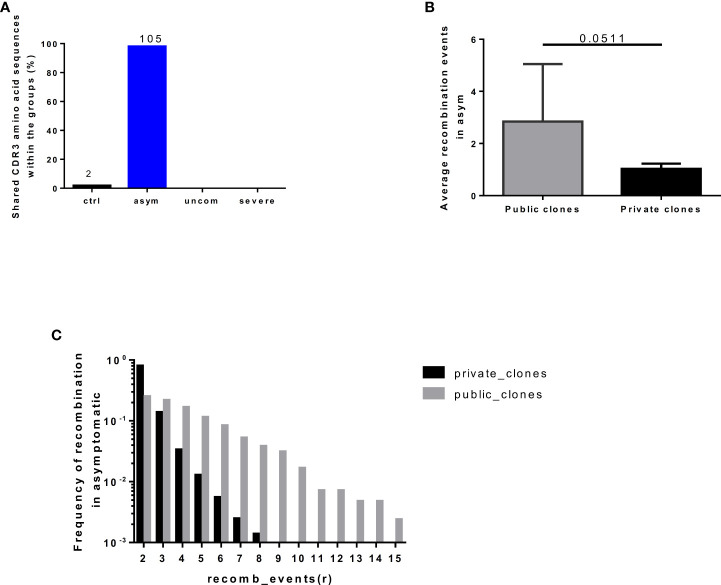
Frequency of recombination events in public and private clones. The number of **(A)** shared clones found in individuals in each group. **(B)** Average recombination events in the asymptomatic group **(C)** Degree of convergent recombination events in the public and private clones occurring in the asymptomatic group at threshold of 2<=r<=15, recombination events. The plot shows the mean and standard deviation. Data presented in boxplot shows inter-quartile ranges with horizontal lines indicating the median.

Public TCRs are generated via convergent recombination [a process whereby the recombination biases produce some TCR sequences more frequently (shared/public) than others]. We determined the degree of convergent recombination that underlies the generation of the public and private TCRs found in our study groups. However, since public TCRs were predominant in the asymptomatic group (**Figure 5A**), we could only compare average recombination events for both public and private TCRs for this group. We found that even though the average number of convergent recombination events generating the CDR3 sequences was elevated within the public TCRs compared to private TCRs there was no significant difference (p=0.0511, **Figure 5B**). Interestingly, studying the recombination events in the asymptomatic group at different thresholds (*r*) of recombination, 2≤ *r* ≤ 15, the frequency of convergent recombination events declines rapidly in private TCRs, reaching 0 at *r*=9 (**Figure 5C**). In contrast, the number of recombination events declines much more slowly in the public TCRs. These observations are consistent with the existence of a greater likelihood of producing public TCRs during V(D)J recombination as well as a greater degree of antigen-driven selection of the same TCRs in the studied asymptomatic individuals.

### Grouping TCR sequences based on their antigenic specificity

It has been shown that TCRs recognizing or responding to the same antigens mostly have similar TCR CDR3 sequences (28). Using the GLIPH (grouping of lymphocytes based on paratope hotspots) algorithm, we interrogated our datasets to determine which sets of TCR sequences have similar antigen specificities. We compiled a list of the top 100 most abundant TCRs from each infected individual and then clustered them using GLIPH. We obtained a total of 1846 specificity clusters. We looked for clusters with four unique TCRs found in at least 3 individuals. There were 9 such clusters containing a total of 46 unique TCRs (**Table 3**). We found a specificity cluster, with the representative CDR3 sequence CASCEADRDTDTQYF, that appears in a much higher fraction of the asymptomatic group (57%) compared to the uncomplicated (22%) and severe (0%) groups. This specificity cluster contains a 4-mer amino acid sequence motif with a DRD nucleus. In contrast, a specificity cluster with the representative CDR3 sequence CASSLEPSRTYNEQFF appears in a much higher fraction of the severe group (75%) compared to the uncomplicated (67%) and asymptomatic (43%) groups.

**Table 3:** TCR specificity clusters observed in the *P. falciparum-*infected cohort.

Specificity cluster	CDR3 clones	Motifs	TRBV	TRBJ	Patient proportion			Freq (mean)	Freq (SD)
					asym(n =7)	uncom (n =9)	seve (n=8)	
CASSLVAVTDTQYF	casslvatqetqyf		V7-8	J2-5	4	3	3	1.63E-05	9.65E-05
	cassliaaqetqyf		V7-8	J2-5				5.45E-05	0.000201
	casslvaaqetqyf		V7-8	J2-5				1.16E-05	5.25E-05
	cassLVAVhneqff	LVAV	V7-8	J2-1				4.65E-05	0.000155
	cassLVAVnyneqff	LVAV	V7-9	J2-1				8.40E-06	4.97E-05
	cassLVAVqetqyf	LVAV	V7-8	J2-5				1.01E-05	4.58E-05
	casslvasqetqyf		V7-8	J2-5				1.05E-05	5.40E-05
	cassLVAVtdtqyf	LVAV	V7-8	J2-3				1.98E-05	0.000106
CASCEADRDTDTQYF*	casslgDRDAdtqyf	DRDA	V7-9	J2-3	4	2	0	0.00011871	0.0007
	csveADRDstdtqyf	ADRD	V29-1	J2-3				1.23E-05	7.25E-05
	castfDRDArneqff	DRDA	V28	J2-1				6.49E-06	3.84E-05
	castADRDypkniqyf	ADRD	V12-3	J2-4				1.78E-05	0.000106
	catsDRDAniqyf	DRDA	V15	J2-4				7.97E-06	4.72E-05
	casceADRDtdtqyf	ADRD	V12-4	J2-3				5.06E-06	2.99E-05
	csarA*DRD*Ayneqff	ADRD, DRDA	V20-1	J2-1				0.00010187	0.000557
CASSLEPSRTYNEQFF	casslG*PSR*Tnneqff	GPSR, PSRT	V7-3	J2-1				4.64E-05	0.0002
	casnlG*PSR*TYneqff	GPRS, PSRT, SRTY	V7-3	J2-1	3	6	6	0.00011161	0.00066
	cassleP*SRT*Yneqff	PSRT, SRTY	V7-3	J2-1				8.28E-06	4.90E-05
	cassmG*PSR*TYneqff	GPRS, PSRT, SRTY	V7-3	J2-1				0.00017257	0.000972
	ctsslG*PSR*TYneqff	GPRS, PSRT, SRTY	V7-3	J2-1				8.28E-06	4.90E-05
	casslG*PSR*TYneqff	GPRS, PSRT, SRTY	V7-3	J2-1				0.01195658	0.032472
CASSLKRNAYEQYF	cassLKRvtnsplhf	LKR	V7-3	J1-6				4.49E-05	0.000266
	csaLKReradtqyf	LKR	V20-1	J2-7	2	1	3	2.49E-06	1.48E-05
	cassLKRdrvasyeqyf	LKR	V4-1	J2-7				0.0001616	0.000708
	cqqLKRggrgdpvf	LKR	V7-2	J2-5				3.80E-07	2.25E-06
	cassLKRnayeqyf	LKR	V27	J2-7				5.64E-05	0.000334
						
CASSQEYSSGGRYEQYF	casSQEYrvfyeqyf	SQEY	V4-1	J2-7				2.07E-05	0.000123
	casSQEYasqpagplhf	SQEY	V4-3	J1-6	2	3	0	5.78E-05	0.000342
	casSQEYrifyeqyf	SQEY	V4-1	J2-7				1.39E-05	8.23E-05
	casSQEYssggryeqyf	SQEY	V4-2	J2-7				0.00016951	0.000711
						
CASSEDRADQPQHF	cassEDRAdqpqhf	EDRA	V12-4	J1-5				8.46E-05	0.000501
	cassqpEDRAntgelff	EDRA	V4-1	J2-2	2	3	0	0.00011343	0.000602
	csvEDRAryeqyf	EDRA	V29-1	J2-7				3.37E-06	2.00E-05
	cassqEDRAyyeqyf	EDRA	V4-1	J2-7				1.42E-05	8.41E-05
CASSKMTGASTDTQYF	casslalMTGAqetqyf	MTGA	V5-1	J2-5				3.37E-06	2.00E-05
	casskMTGAstdtqyf	MTGA	V3-1	J2-3	1	2	1	5.41E-05	0.000306
	casssyMTGAdteaff	MTGA	V5-1	J1-1				1.75E-05	0.000104
	cassqMTGAstdtqyf	MTGA	V4-1	J2-3				7.45E-05	0.000401
CSASLIGWNGGEQFF	cassypgtNGGElff	NGGE	V6-5	J2-2				1.62E-05	9.59E-05
	csaslfgW*NGG*Eqff	WNGG, NGGE	V20-1	J2-1	1	4	2	0.00130294	0.004429
	csasligW*NGG*Eqff	WNGG, NGGE	V20-1	J2-1				3.09E-05	0.000183
	csasifgW*NGG*Eqff	WNGG, NGGE	V20-1	J2-1				2.61E-05	0.000155
CASVPDWGTGELFF	csaPDWsneqff	PDW	V20-1	J2-1				0.00019368	0.000773
	casvPDWgtgelff	PDW	V6-1	J2-2	1	3	2	2.49E-05	0.000147
	csaPDWsygvgeqyf	PDW	V20-1	J2-7				1.23E-05	7.30E-05
	csaPDWnneqff	PDW	V20-1	J2-1				0.0001142	0.000449

Red coloured letters indicate predicted binding motifs with 10 fold enrichment of probability< 0.001. The asterisk (*) indicate specificity cluster that significantly occur (p<0.05) at unequal distributions in the groups.

We aligned these specificity clusters to TCR CDR3 sequences found in VDJdb (29) to determine if any of their representative sequences have previously been associated with other human infections. We found two TCR CDR3 sequences CSAPDWNNEQFF (same sequence) and CSAPDWSNEQFF (with one amino acid mismatch) from the cluster CASVPDWGTGELFF with the PDW motif to have previously been identified CMV infection. In CMV, these TCRs are known to interact with the CRVLCCYVL epitope of the IE-1 antigen, which is an MHC Class I allele C restricted antigen (HLA-C*07:02) (39). Additionally, we identified another CDR3 sequence (CSVEDRARYEQYF) from our dataset that is almost identical to an epitope specific CD8+ TCR CDR3 sequence that has been associated with the response to HIV-1 infection (CSVEDRANEQYF) (40).

## Discussion

The role of T cells is crucial in malaria infection, with dynamic changes occurring in both CD4 and CD8 T cell activity in response to the pathogen, in both human and animal models (41). Usually, the activity of malaria-specific T cells is measured using approaches such as ELISPOT and flow cytometry. Studying human malaria-specific T cells through next-generation sequencing approach to obtain information such as the diversity of the TCR repertoire, gene usage, convergent recombination events and grouping them into antigen specificities has never been performed. However, this information is necessary to help identify and target antigen-specific responses in malaria infections to improve the performance of malaria vaccine candidates and potentially identify promising therapeutic agents. Therefore, in this preliminary study, we used 5’RACE amplification and bulk TCRβ sequencing to characterize the dynamic changes occurring during *P. falciparum* malaria infection in a cohort of children with asymptomatic, uncomplicated and severe disease. We show that TCR repertoire within the malaria-infected group differs in diversity, clonality, TCR sharing and gene segment usage. In addition, the diversity of the repertoire is inversely correlated with parasitemia. Interestingly, TCR sharing was very high in the asymptomatic cohort, but comparatively negligible in the uncomplicated and severe malaria groups.

The overview of a TCR repertoire may be inferred from the CDR3 sequence length distribution since the biases observed in the length distribution can be driven by exposure to specific antigens resulting in the selection of specific T cells probably with increased avidity.

Thymic selection shapes the CDR3 distribution, including CDR3 sequence lengths, and the specificity of the TCRs for antigens (42). The length of the CDR3 sequence may affect the folding of the TCR loop (33), which may result in a conformation change and thus alter the TCR’s interaction with MHC molecules (43). Shorter CDR3 lengths have been associated with pathologic outcomes in autoimmune diseases such as ulcerative colitis (44) and diabetes (12, 45). Previous studies in mice have suggested a role for pathogenic T cells in the development of severe malaria (cerebral malaria) under the inflammation hypothesis (46, 47). These pathogenic T cells have been restricted to those expressing the Vβ 8.1 (TRBV 12) TCR gene segment (19, 20, 22, 48). In this study, we found shorter CDR3 lengths in the malaria-infected group compared to the healthy controls (despite the low number of productive sequences), an intriguing observation that deserves further study. Additionally, it has been reported that TCR amino acid sequences that are shared tend to be shorter (49), therefore, the shorter CDR3 lengths observed in the malaria-infected group could denote that these cells have been clonally expanded to respond to a similar antigen.

The usage of Vβ and Jβ gene segments has been described to be non-uniform in healthy controls. The extent of repertoire skewing we found within the malaria-infected group varied significantly, with clonal dominance observed in specific T cell clones. The preferential use of certain TCR Vβ and Jβ gene segments could result from *P falciparum* antigen stimulation leading to the expansion of specific T cell clones. Likewise, the selective usage of gene segments may be influenced by the severity of the infection or disease. Also, the segments which were present in all infected groups could reflect gene segments selection by a common antigen present in all the infected groups. These observations are consistent with the view that malaria infection influences the TCR repertoire resulting in the activation and expansion of *P. falciparum*-responsive T cell clones. Delineating the immune response from these specific clones may help in identifying the antigens stimulating the activation and expansion of such specific TCRs. Within the infected group, we observed increased diversity in the asymptomatic group compared to uncomplicated and severe groups. Because a more diverse repertoire is better able to protect against a large diversity of pathogen-derived antigens, this observation is consistent with the absence of symptoms in the asymptomatic group (50, 51). The decreased diversity in the uncomplicated and severe malaria group could be as a result of oligoclonal T cell responses occurring during infection. It could also be that the low parasitemia levels found in the asymptomatic group was too low to affect the TCR diversity. Likewise, the higher parasitemia levels found in the diseased groups might have caused T cell increased activation, exhaustion and apoptosis (5, 23), events which are likely to decrease TCR diversity as observed in the diseased groups.

The frequency and development of public TCR CDR3 sequences are mostly predicted during convergent recombination and germline somatic recombination (52). Also, it can be affected by the TCR affinity for peptide-MHC complexes (53). Public TCRs have been proposed to be crucial for preservation of tolerance to self-antigens (54), shared to respond to same pathogen (55). Importantly, public TCRs have been associated with favorable outcomes from disease (56) as well as immune escape (57). Public TCR clones may be expanded during antigen stimulation either *via* an infection or vaccination (58, 59). Within the infected group, only the asymptomatic group exhibited a substantial degree of TCR sharing. In contrast, TCR sharing was rare in the uncomplicated and severe groups. This was intriguing since increased public TCR have mostly been associated with good clinical outcomes (37). This was quite interesting, since previously, we have been able to establish that asymptomatic infections in these cohorts are characterized by a lower T reg activation and T cell activation phenotypes compared to the clinical malaria group (5). This may be due to exposure to the large antigenic repertoire of *P. falciparum* antigens, coupled with diversity and polymorphisms of the parasite. In addition, public T cell responses have been found to be cross-reactive (58), therefore, providing for an improved protection against disease. It is possible that a better understanding of the mechanisms governing TCR sharing will inform in the development of improved pharmaceutical interventions against malaria.

TCRβ sequences with same specificity tend to harbor short sequence motifs which are conserved (60) and predicted to interact with antigenic peptides (28). We found multiple groups of TCR sequences predicted to have similar antigen specificities in our infected groups. Interestingly, some specificity groups were more prevalent in the symptomatic group compared to the other infected groups. However, we could not determine if these specificity clusters were HLA specific. Interestingly certain TCR CDR3 sequences that we predicted to be selected during malaria infection were previously associated with other infectious diseases such as CMV and HIV, consistent with their publicness. It will be fascinating if these CDR3β similarity clusters can be validated since this has the potential to aid in the identification of antigenic peptides to aid in vaccine development (61).

This study has a number of limitations. The lower TCR diversity in the healthy controls was quite unexpected, since the median RNA Integrity Number measured for the extracted RNA used for sequencing was 6.3 for the controls. Apart from the lower sample size, nutritional status, which could have also impacted TCR repertoire, was not determined (62). Importantly, the controls were not screened for other infectious diseases such as chronic helminth-infections which are popular in children of school going age (63, 64) in the region. it will be interesting if prospective and longitudinal studies are designed with increased sample size, multiple sampling points, such as before and after antimalarial treatment in infected patients and different transmission seasons to track the dynamics of T cell development in *P. falciparum* infection.

Moreover, we have initially profiled the frequency of activated Tregs and T cell activation markers in this cohort before and after stimulation with infected RBCs (iRBC) as well as determined their impact on parasitemia control (5). Before, iRBC stimulation, we observed that asymptomatic infections were characterized by lower levels of T regs and Treg activation which corresponded with decreased T cell activation and low parasitemia. However, after stimulation, we observed an increase in the T cell activation. Therefore, we proposed that children with asymptomatic malaria may have a larger latent repertoire of parasite responsive T cells. Also, we profiled the frequency of T cell inhibitory and senescent markers in these cohorts (23) and observed an increased expression of T cell inhibitory and senescent markers in children with clinical malaria which may contribute to the poor immunity or the lack of sterile immunity to malaria despite years of continuous exposure.

In conclusion, our preliminary data show that the T cell repertoire during *P. falciparum* infection sustains significant perturbations, characterized by a decrease in TCR diversity, the selective expansion of specific clonotypes and V-J gene segments, and the downregulation of public T cells in children in poorer outcomes (uncomplicated and severe) compared to asymptomatic children. This indicates that the TCR repertoire may serve as a useful biomarker in understanding protective immunity to *P. falciparum* infections.

## Data availability statement

The data presented in the study are deposited in the Gene Expression Omnibus (GEO) repository, accession number GSE212894.

## Ethics statement

The studies involving human participants were reviewed and approved by Institutional Review Board of the Noguchi Memorial Institute for Medical Research, University of Ghana. Written informed consent to participate in this study was provided by the participants’ legal guardian/next of kin.

## Author contributions

WN and AF conceived the idea. WN, AF and MO designed the experiments. EK-B and JQ assisted with sampling. NN assisted with the sequencing. WN and AF performed the sequencing and all other data analysis. AD and BG assisted with the sequencing analysis. WN, MO and KK supervised the work. AF and WN wrote the paper with contributions from co-authors. All authors contributed to the article and approved the submitted version.

## Funding

AF was supported by a PhD fellowship from the World Bank African Centres of Excellence Grant (ACE02-WACCBIP: Awandare). This work was supported by the International Development Research Center research grant from the African Institute for Mathematical Sciences, Ghana, the L’Oreal-UNESCO for Women in Science Grant (AF) and the Carnegie Corporation of New York and the University of Ghana BanGA Ph.D. Research Grant (AF).

## Acknowledgments

The authors thank the children who participated in this study as well as their parents and guardians. We are also grateful to the Directors and Clinical staff at the Shai-Osudoku Health Directorate and the Hohoe municipal hospital.

## Conflict of interest

Author NN is employed by MIODX Inc, San Jose, California, USA.

The remaining authors declare that the research was conducted in the absence of any commercial or financial relationships that could be construed as a potential conflict of interest.

## Publisher’s note

All claims expressed in this article are solely those of the authors and do not necessarily represent those of their affiliated organizations, or those of the publisher, the editors and the reviewers. Any product that may be evaluated in this article, or claim that may be made by its manufacturer, is not guaranteed or endorsed by the publisher.

## Supplementary material

Supplementary Figure 1 Frequency of V and J gene usage after bootstrap re-sampling. The data show significant **(A-F)** V gene between **(A)** control and asymptomatic **(B)** control and uncomplicated **(C)** control and severe, **(D)** asymptomatic and uncomplicated, **(E)** asymptomatic and severe, **(F)** uncomplicated and severe malaria groups;.g-l) J gene usage between **(G)** control and asymptomatic **(H)** control and uncomplicated **(I)** control and severe, **(J)** asymptomatic and uncomplicated, **(K)** asymptomatic and severe, **(L)** uncomplicated and severe malaria groups after bootstrapping (10^5^) with non-parametric test for significance. The bars show the mean proportions with the standard error. Benjamin-Hochberg test was used to correct for multiple comparisons.

Supplementary Figure 2 The Kulback-Leibler divergence for TRBV gene segment usage compared between the **(A)** controls with the asymptomatic, uncomplicated and severe malaria cases; **(B)** asymptomatic with the uncomplicated and severe malaria cases; **(C)** uncomplicated and severe malaria cases. These were generated after 10^4^ iterations.

Supplementary Figure 3 A heatmap representation of the gene recombination of the TRBV and TRBJ genes classifying the study groups (59 TRBV × 13 TRBJ) is shown.

Supplementary Figure 4 The number of recombination events at different r values compared between the study population, P<0.05 was considered statistically significant. Ctrl = control (n=4); asym = asymptomatic(n=7); uncom = uncomplicated (n=9); and severe malaria (n=8).
