# Supplementary material for: Perturbations in the T cell receptor β repertoire during malaria infection in children: A preliminary study
Source: Front Immunol. 2022 Oct 13;13:971392. doi: 10.3389/fimmu.2022.971392 (PMC9606469; doi:10.3389/fimmu.2022.971392)
Supplement: Supplementary Figure 1 — Frequency of V and J gene usage after bootstrap re-sampling. The data show significant (A-F) V gene between (A) control and asymptomatic (B) control and uncomplicated (C) control and severe, (D) asymptomatic and uncomplicated, (E) asymptomatic and severe, (F) uncomplicated and severe malaria groups;.g-l) J gene usage between (G) control and asymptomatic (H) control and uncomplicated (I) control and severe, (J) asymptomatic and uncomplicated, (K) asymptomatic and severe, (L) uncomplicated and severe malaria groups after bootstrapping (105) with non-parametric test for significance. The bars show the mean proportions with the standard error. Benjamin-Hochberg test was used to correct for multiple comparisons. [file DataSheet_1.pdf]

a.

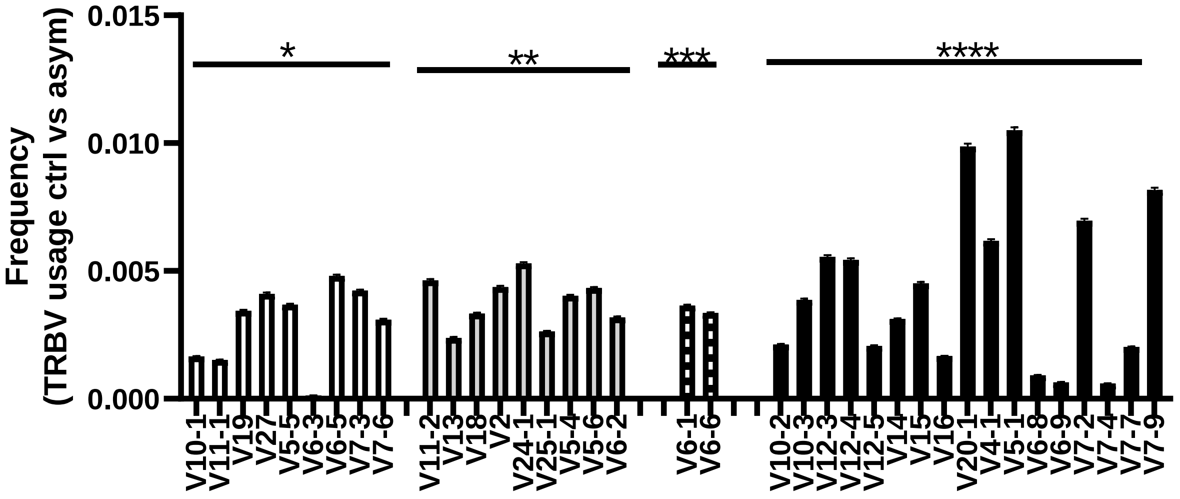

Frequency
(TRBV usage ctrl vs asym)
0.015
0.010
0.005
0.000
*
**
***
****
V10-1
V11-1
V19
V27
V5-5
V6-3
V6-5
V7-3
V7-6
V11-2
V13
V18
V2
V24-1
V25-1
V5-4
V5-6
V6-2
V6-1
V6-6
V10-2
V10-3
V12-3
V12-4
V12-5
V14
V15
V16
V20-1
V4-1
V5-1
V6-8
V6-9
V7-2
V7-4
V7-7
V7-9

b.

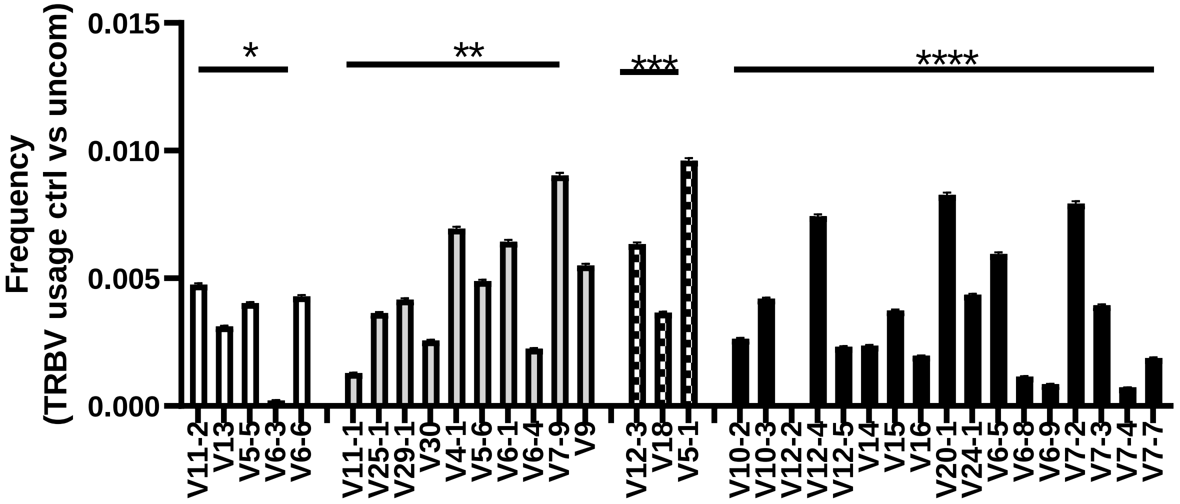

Frequency
(TRBV usage ctrl vs uncom)
0.015
0.010
0.005
0.000
*
**
***
****
V11-2
V13
V5-5
V6-3
V6-6
V11-1
V25-1
V29-1
V30
V4-1
V5-6
V6-1
V6-4
V7-9
V9
V12-3
V18
V5-1
V10-2
V10-3
V12-2
V12-4
V12-5
V14
V15
V16
V20-1
V24-1
V6-5
V6-8
V6-9
V7-2
V7-3
V7-4
V7-7

c.

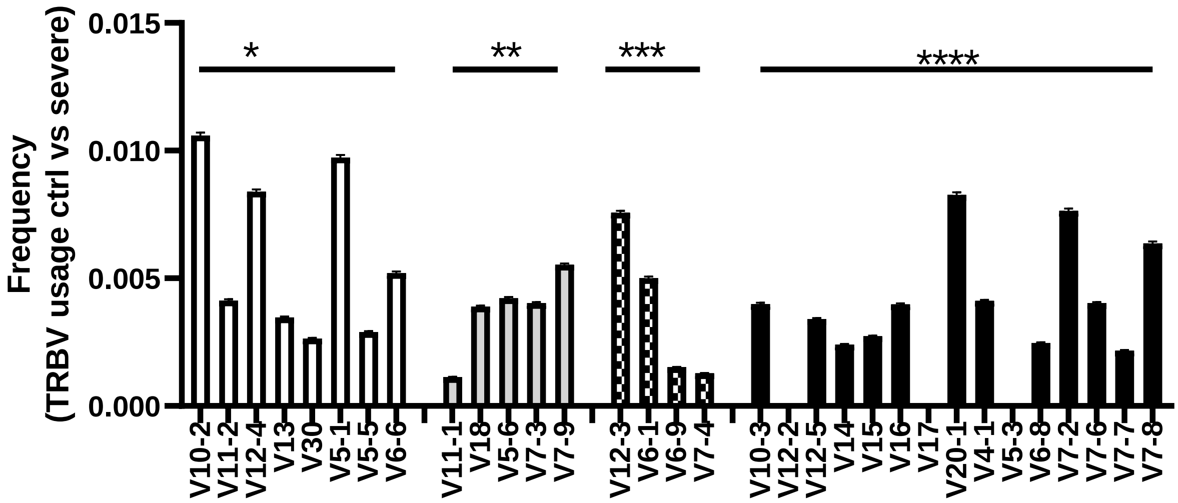
Frequency
(TRBV usage ctrl vs severe)
0.015
0.010
0.005
0.000
*
**
***
****
V10-2
V11-2
V12-4
V13
V30
V5-1
V5-5
V6-6
V11-1
V18
V5-6
V7-3
V7-9
V12-3
V6-1
V6-9
V7-4
V10-3
V12-2
V12-5
V14
V15
V16
V17
V20-1
V4-1
V5-3
V6-8
V7-2
V7-6
V7-7
V7-8

d.

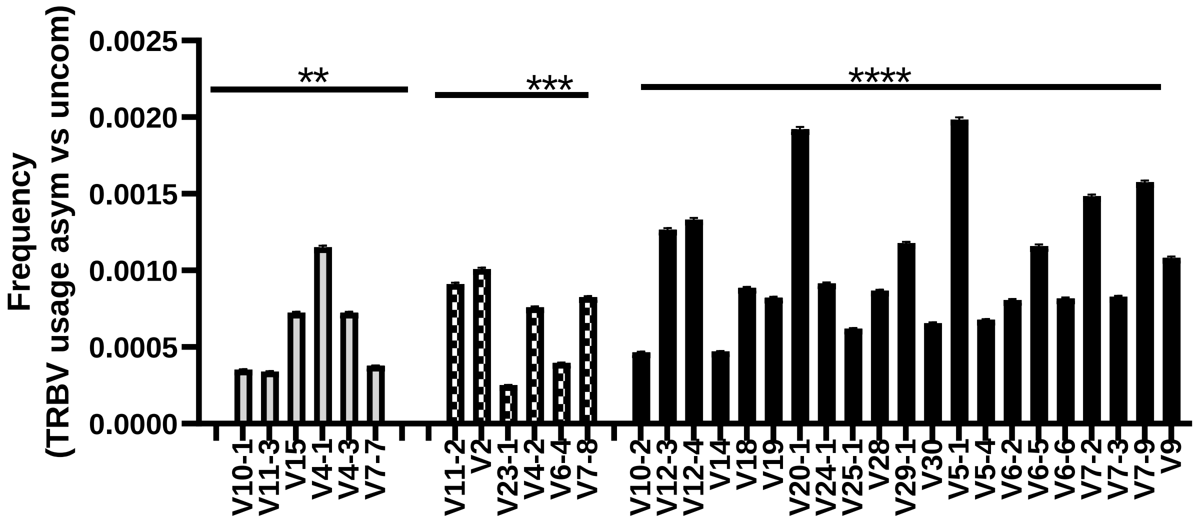
Frequency
(TRBV usage asym vs uncom)
0.0025
0.0020
0.0015
0.0010
0.0005
0.0000
**
***
****
V10-1
V11-3
V15
V4-1
V4-3
V7-7
V11-2
V2
V23-1
V4-2
V6-4
V7-8
V10-2
V12-3
V12-4
V14
V18
V19
V20-1
V24-1
V25-1
V28
V29-1
V30
V5-1
V5-4
V6-2
V6-5
V6-6
V7-2
V7-3
V7-9
V9

e.

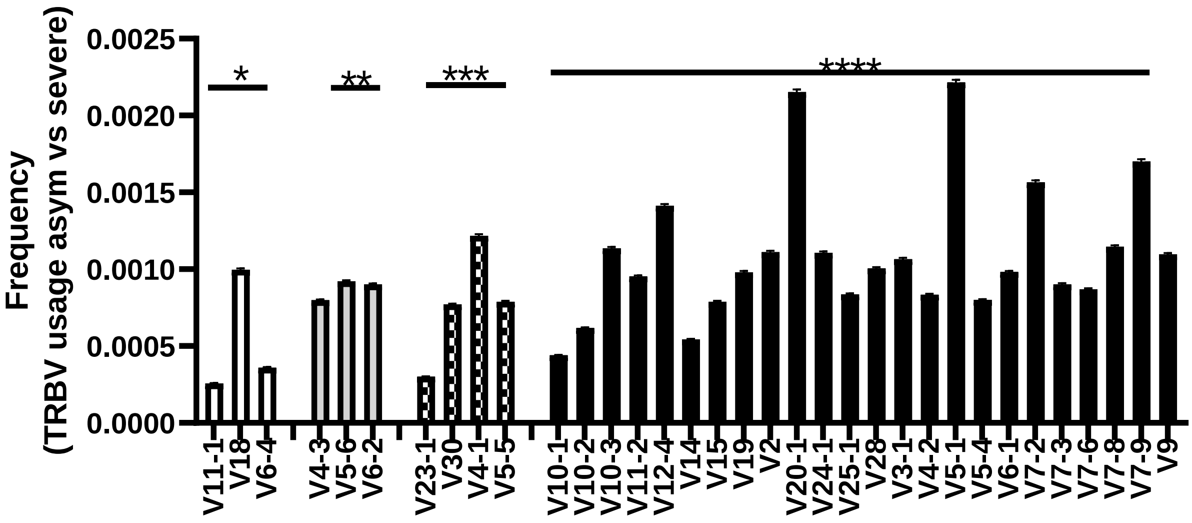
Frequency
(TRBV usage asym vs severe)
0.0025
0.0020
0.0015
0.0010
0.0005
0.0000
*
**
***
****
V11-1
V18
V6-4
V4-3
V5-6
V6-2
V23-1
V30
V4-1
V5-5
V10-1
V10-2
V10-3
V11-2
V12-4
V14
V15
V19
V2
V20-1
V24-1
V25-1
V28
V3-1
V4-2
V5-1
V5-4
V6-1
V7-2
V7-3
V7-6
V7-8
V7-9
V9

f.

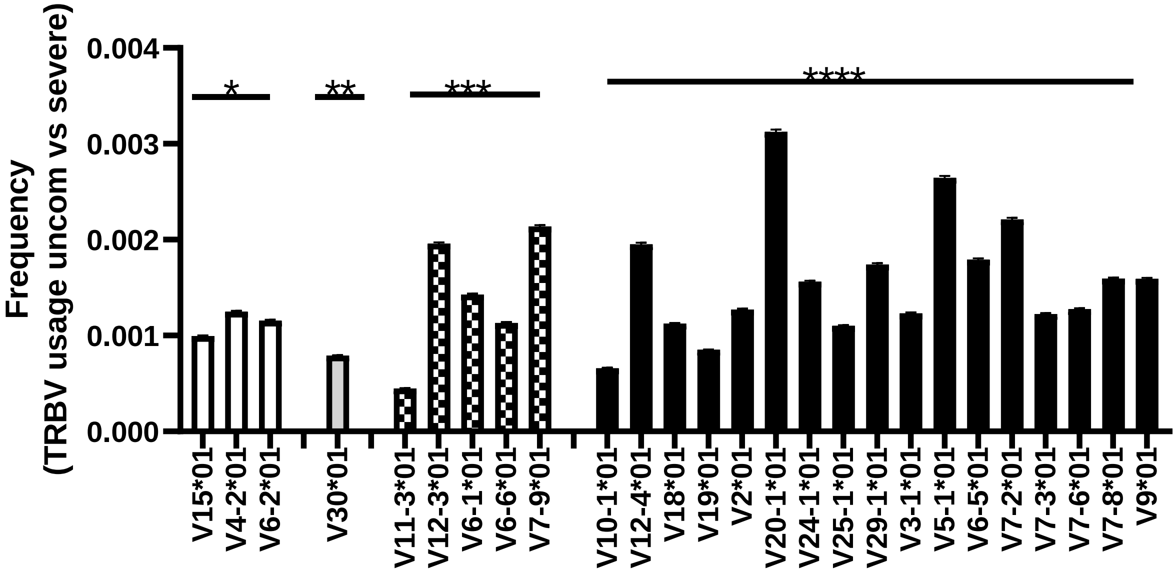
Frequency
(TRBV usage uncom vs severe)
0.004
0.003
0.002
0.001
0.000
*
**
***
****
V15*01
V4-2*01
V6-2*01
V30*01
V11-3*01
V12-3*01
V6-1*01
V6-6*01
V7-9*01
V10-1*01
V12-4*01
V18*01
V19*01
V2*01
V20-1*01
V24-1*01
V25-1*01
V29-1*01
V3-1*01
V5-1*01
V6-5*01
V7-2*01
V7-3*01
V7-6*01
V7-8*01
V9*01

g.

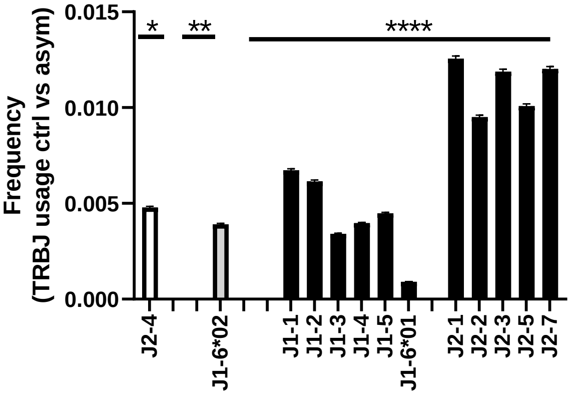
Frequency
(TRBJ usage ctrl vs asym)
0.015
0.010
0.005
0.000
*
**
****
J2-4
J1-6*02
J1-1
J1-2
J1-3
J1-4
J1-5
J1-6*01
J2-1
J2-2
J2-3
J2-5
J2-7

h.

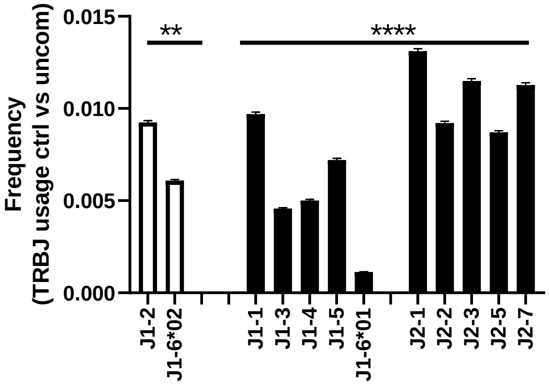
Frequency
(TRBJ usage ctrl vs uncom)
0.015
0.010
0.005
0.000
**
****
J1-2
J1-6*02
J1-1
J1-3
J1-4
J1-5
J1-6*01
J2-1
J2-2
J2-3
J2-5
J2-7

i.

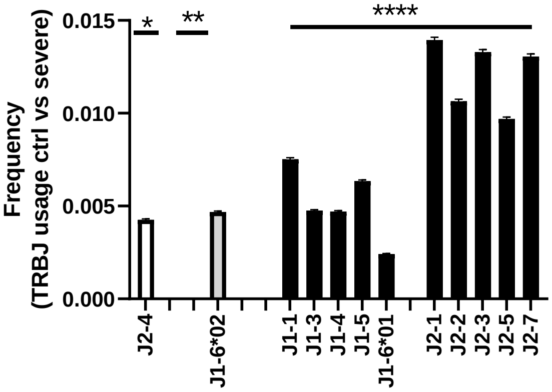
Frequency
(TRBJ usage ctrl vs severe)
0.015
0.010
0.005
0.000
*
**
****
J2-4
J1-6*02
J1-1
J1-3
J1-4
J1-5
J1-6*01
J2-1
J2-2
J2-3
J2-5
J2-7

j.

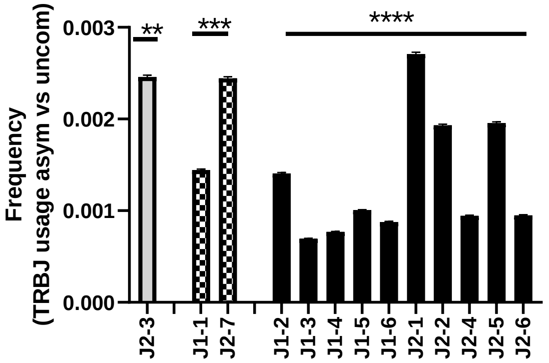
Frequency
(TRBJ usage asym vs uncom)
0.003
0.002
0.001
0.000
**
***
****
J2-3
J1-1
J2-7
J1-2
J1-3
J1-4
J1-5
J1-6
J2-1
J2-2
J2-4
J2-5
J2-6

k.

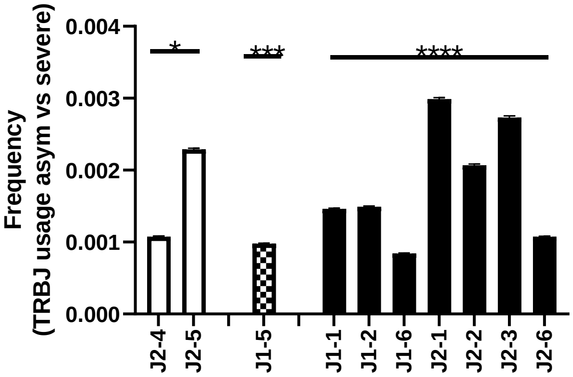
Frequency
(TRBJ usage asym vs severe)
0.004
0.003
0.002
0.001
0.000
*
***
****
J2-4
J2-5
J1-5
J1-1
J1-2
J1-6
J2-1
J2-2
J2-3
J2-6

l.

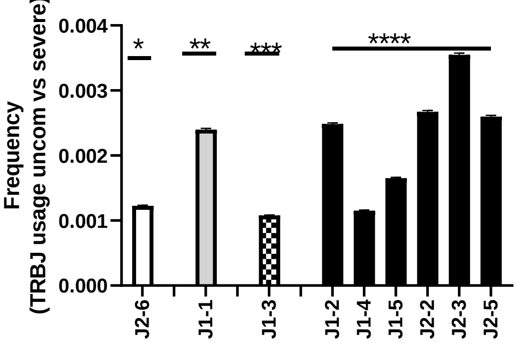
Frequency
(TRBJ usage uncom vs severe)
0.004
0.003
0.002
0.001
0.000
*
**
***
****
J2-6
J1-1
J1-3
J1-2
J1-4
J1-5
J2-2
J2-3
J2-5

Supplementary Figure 1. **Frequency of V and J gene usage after bootstrap re-sampling**. The data show significant (**a-f**) V gene between (**a**) control and asymptomatic (**b**) control and uncomplicated (**c**) control and severe, (**d**) asymptomatic and uncomplicated, (**e**) asymptomatic and severe, (**f**) uncomplicated and severe malaria groups; .**g-l**) J gene usage between (**g**) control and asymptomatic (**h**) control and uncomplicated (**i**) control and severe, (**j**) asymptomatic and uncomplicated, (**k**) asymptomatic and severe, (**l**) uncomplicated and severe malaria groups after bootstrapping (10^5) with non-parametric test for significance. The bars show the mean proportions with the standard error. Benjamin-Hochberg test was used to correct for multiple comparisons.

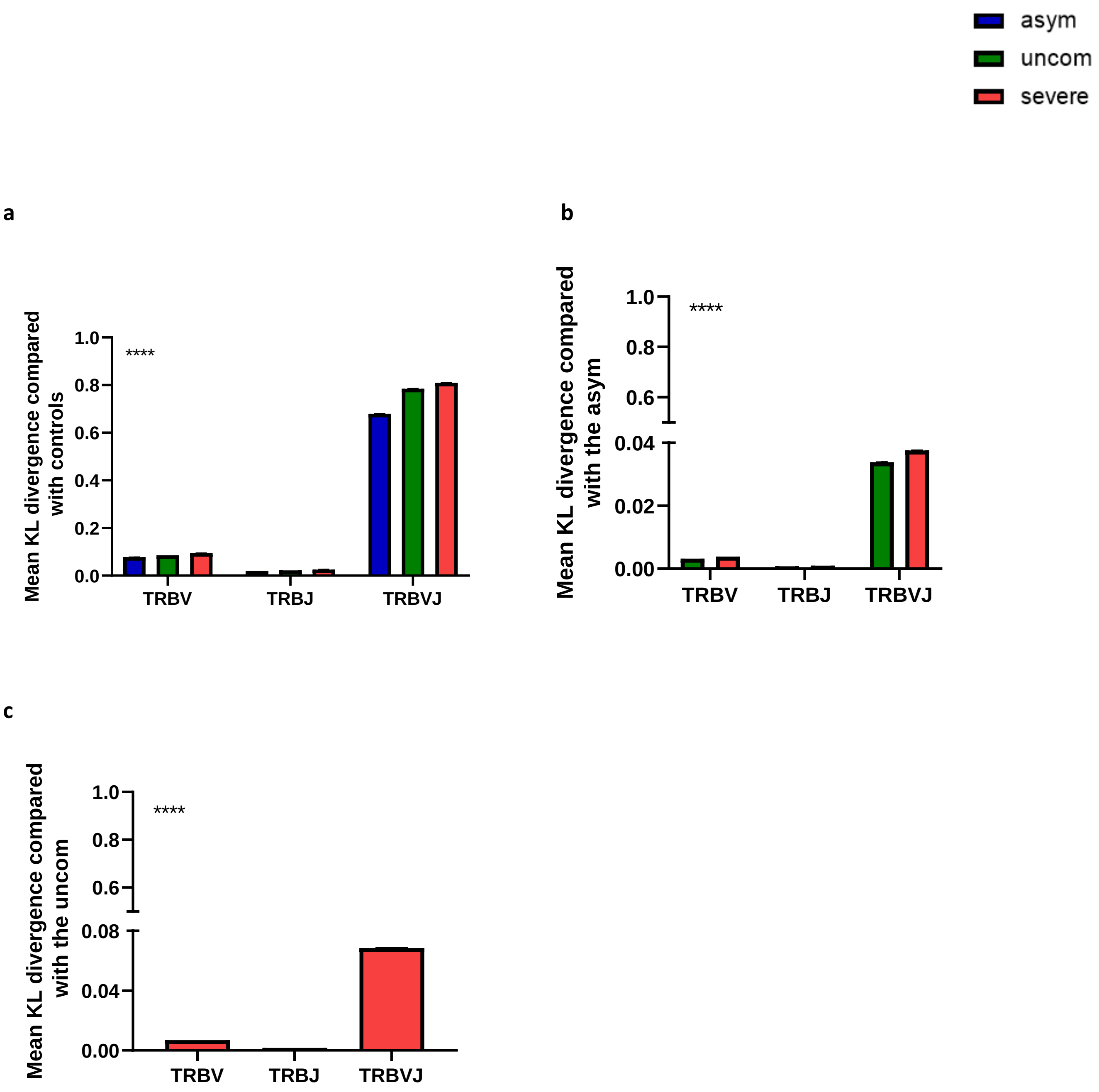

Supplementary Figure 2: **The Kulback-Leibler divergence for TRBV gene segment usage.**

The divergence was compared between the a) controls with the asymptomatic, uncomplicated

and severe malaria cases; b) asymptomatic with the uncomplicated and severe malaria cases; c) uncomplicated and severe malaria cases. These were generated after 10^4 iterations.

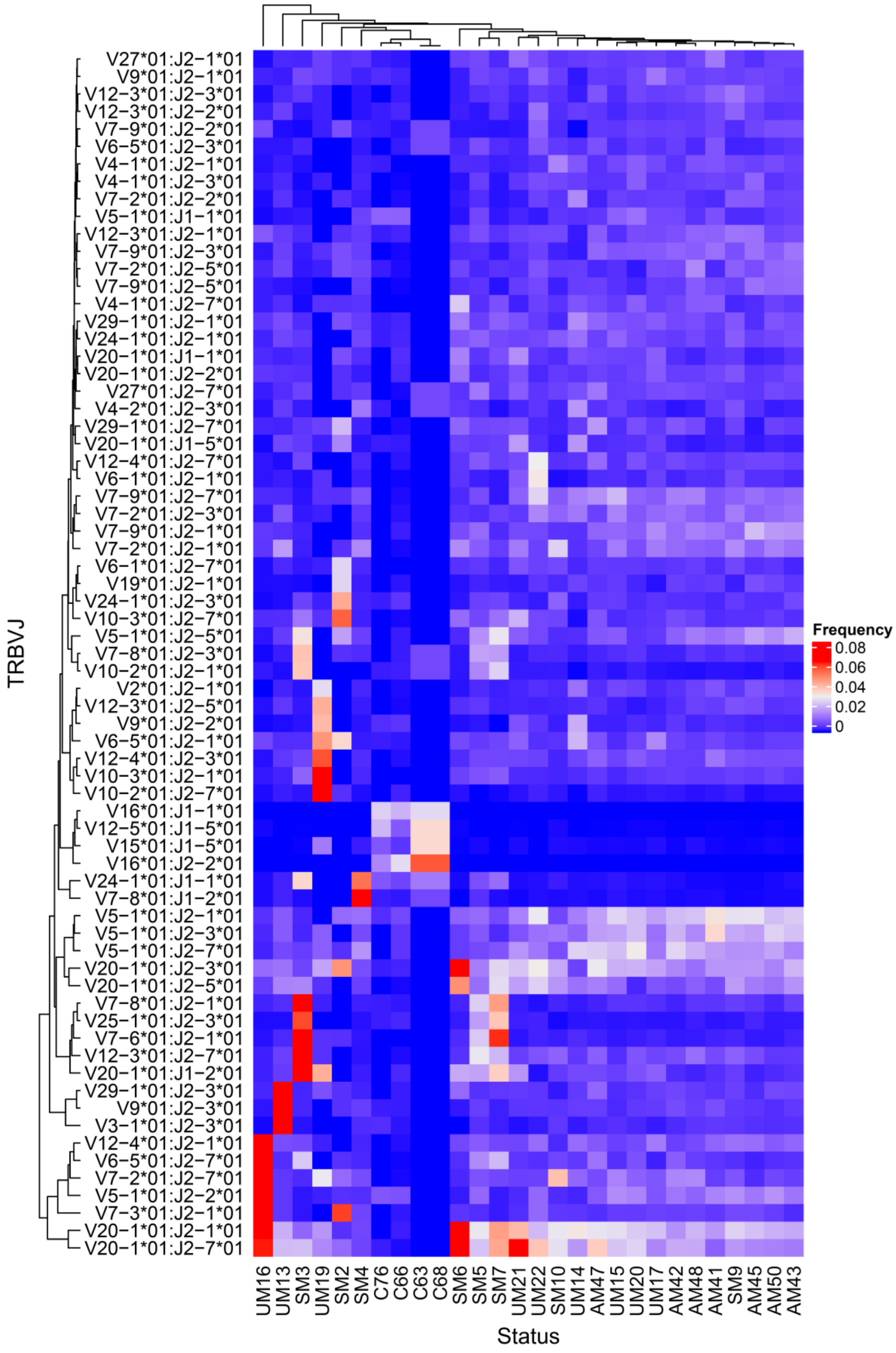

TRBVJ
V27*01:J2−1*01
V9*01:J2−1*01
V12−3*01:J2−3*01
V12−3*01:J2−2*01
V7−9*01:J2−2*01
V6−5*01:J2−3*01
V4−1*01:J2−1*01
V4−1*01:J2−3*01
V7−2*01:J2−2*01
V5−1*01:J1−1*01
V12−3*01:J2−1*01
V7−9*01:J2−3*01
V7−2*01:J2−5*01
V7−9*01:J2−5*01
V4−1*01:J2−7*01
V29−1*01:J2−1*01
V24−1*01:J2−1*01
V20−1*01:J1−1*01
V20−1*01:J2−2*01
V27*01:J2−7*01
V4−2*01:J2−3*01
V29−1*01:J2−7*01
V20−1*01:J1−5*01
V12−4*01:J2−7*01
V6−1*01:J2−1*01
V7−9*01:J2−7*01
V7−2*01:J2−3*01
V7−9*01:J2−1*01
V7−2*01:J2−1*01
V6−1*01:J2−7*01
V19*01:J2−1*01
V24−1*01:J2−3*01
V10−3*01:J2−7*01
V5−1*01:J2−5*01
V7−8*01:J2−3*01
V10−2*01:J2−1*01
V2*01:J2−1*01
V12−3*01:J2−5*01
V9*01:J2−2*01
V6−5*01:J2−1*01
V12−4*01:J2−3*01
V10−3*01:J2−1*01
V10−2*01:J2−7*01
V16*01:J1−1*01
V12−5*01:J1−5*01
V15*01:J1−5*01
V16*01:J2−2*01
V24−1*01:J1−1*01
V7−8*01:J1−2*01
V5−1*01:J2−1*01
V5−1*01:J2−3*01
V5−1*01:J2−7*01
V20−1*01:J2−3*01
V20−1*01:J2−5*01
V7−8*01:J2−1*01
V25−1*01:J2−3*01
V7−6*01:J2−1*01
V12−3*01:J2−7*01
V20−1*01:J1−2*01
V29−1*01:J2−3*01
V9*01:J2−3*01
V3−1*01:J2−3*01
V12−4*01:J2−1*01
V6−5*01:J2−7*01
V7−2*01:J2−7*01
V5−1*01:J2−2*01
V7−3*01:J2−1*01
V20−1*01:J2−1*01
V20−1*01:J2−7*01
UM16
UM13
SM3
UM19
SM2
SM4
C76
C66
C63
C68
SM6
SM5
SM7
UM21
UM22
SM10
UM14
AM47
UM15
UM20
UM17
AM42
AM48
AM41
SM9
AM45
AM50
AM43
Status
Frequency
0.08
0.06
0.04
0.02
0

Supplementary Figure 3: A heatmap representation of the gene recombination of the TRBV and TRBJ genes classifying the study groups (59 TRBV × 13 TRBJ) is shown.

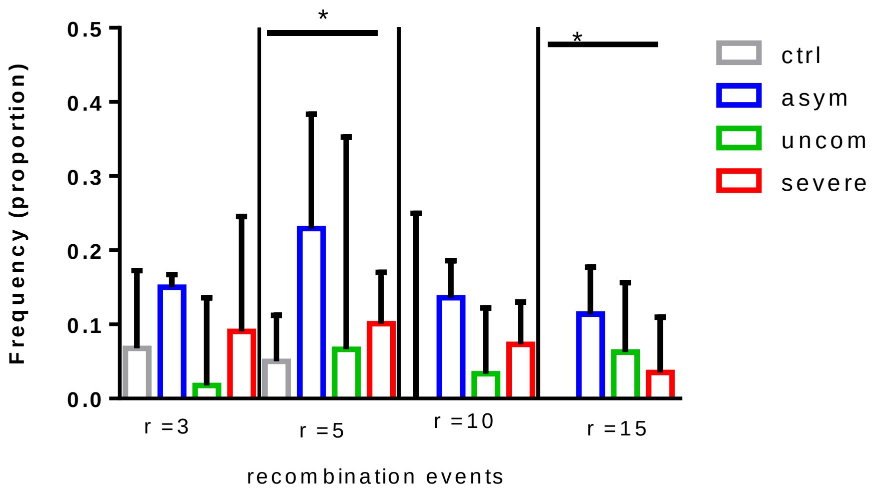

Supplementary Figure 4: **Recombination events in the asymptomatic at different thresholds.** The number of recombination events at different r values compared between the study population, P<0.05 was considered statistically significant. Ctrl = aparasitemic control; asym = asymptomatic; uncom = uncomplicated.
